# Case report of an aggressive cardiac myxofibrosarcoma with rapid disease progression

**DOI:** 10.1093/ehjcr/ytaf447

**Published:** 2025-09-16

**Authors:** Koichi Nakamura, Osamu Kurihara, Nobuaki Kobayashi, Yasuhiro Kawase, Kuniya Asai

**Affiliations:** Cardiovascular Medicine, Nippon Medical School Chiba Hokusoh Hospital, 1715 Kamakari, Inzai, Chiba 270-1694, Japan; Cardiovascular Medicine, Nippon Medical School Chiba Hokusoh Hospital, 1715 Kamakari, Inzai, Chiba 270-1694, Japan; Cardiovascular Medicine, Nippon Medical School Chiba Hokusoh Hospital, 1715 Kamakari, Inzai, Chiba 270-1694, Japan; Cardiovascular Surgery, Nippon Medical School Chiba Hokusoh Hospital, 1715 Kamakari, Inzai, Chiba 270-1694, Japan; Department of Cardiovascular Medicine, Nippon Medical School, 1-1-5 Sendagi, Bunkyo-ku, Tokyo 113-8603, Japan

**Keywords:** Primary cardiac myxofibrosarcoma, Brain metastases, Tumour, Resection, Case report

## Abstract

**Background:**

Primary cardiac myxofibrosarcoma is an exceptionally rare malignant tumour, often asymptomatic until local invasion or distant metastasis occurs. Early diagnosis is challenging, and the prognosis is generally poor owing to high local recurrence and metastasis rates.

**Case Summary:**

We report the case of a 79-year-old woman who presented with neurological symptoms and multiple brain metastases. Imaging showed a broad-based exophytic mass obstructing the left pulmonary vein and extending into the left atrium. Surgical resection was conducted, and the diagnosis of myxofibrosarcoma was confirmed from histology. Despite surgery, the tumour recurred locally within days, and the brain metastases progressed. Considering the patient’s deteriorating condition, no further treatment was initiated. She was then transferred to hospice care and died shortly thereafter.

**Discussion:**

Primary cardiac myxofibrosarcoma is an extremely rare malignancy with non-specific symptoms and poor prognosis owing to its aggressive biological behaviour. In this case, multimodal imaging showed features suggestive of malignancy, including an exophytic mass with a broad attachment base and heterogeneous signal intensity. Despite surgical resection, the tumour recurred rapidly, and brain metastases progressed. The tumour size, high histological grade, and delayed intervention are likely to contribute to poor clinical outcomes. Owing to their rarity, there are no standardized treatment guidelines for primary cardiac sarcomas. In this case, we reconfirmed the high-grade malignancy of the cardiac sarcoma through multiple computerized tomography scans conducted over a short period. Currently, the only method for prompt evaluation using multiple imaging modalities involves early surgical resection combined with adjuvant therapy to improve clinical outcomes.

Learning points

- Primary cardiac myxofibrosarcoma is a rare, aggressive tumour that often stays asymptomatic until late stages and has a high rate for local recurrence and metastasis.
- Multimodal imaging, including transoesophageal echocardiography, cardiac magnetic resonance imaging, and computed tomography, is vital for detecting tumour origin, extent, and malignant features like rapid growth and heterogeneous signal intensity.
- Rapid tumour progression in imaging shows the need for timely multimodal evaluation in patients with distant metastases, though the best treatment strategy remains unclear.

## Introduction

Histopathology reports show approximately 25% of primary cardiac tumours are malignant, with 75%–95% sarcomas.^1^ Primary cardiac myxofibrosarcoma often remains asymptomatic until local invasion or distant metastasis occurs. Surgical resection is typically conducted when feasible.^2^ A definitive diagnosis is established using biopsy and immunohistochemistry.^3^ Myxofibrosarcoma is a highly aggressive, with local recurrence and distant metastasis rates up to 42.9% and 19.0%, respectively, contributing to poor prognosis.^4^ We report the case of a 79-year-old woman with cardiac myxofibrosarcoma and multiple brain metastases at presentation, with early local recurrence shortly after surgical extirpation.

## Summary figure

**Table:** 

Timeline	Clinical events
Date of submission/Day 1	A 79-year-old woman presented with left-sided weakness and visual disturbances. Brain MRI revealed multiple enhancing lesions in both parietal and occipital lobes, suggestive of metastases.
Day 4	Chest computed tomography (CT) identified a mass lesion extending from the left pulmonary vein towards the left atrium.
Day 18	Transthoracic echocardiography did not detect any definitive abnormalities.
Day 21	Transoesophageal echocardiography identified a heterogeneous 40 × 25 mm mass obstructing the left pulmonary vein and partially protruding into the left atrium.
Day 24	Cardiac MRI showed signal characteristics consistent with tumour necrosis and haemorrhage, supporting the suspicion of malignancy.
Day 35	Follow-up cardiac CT demonstrated interval growth of a broad-based exophytic mass further obstructing the left pulmonary vein.
Day 57	Surgical resection was undertaken. Histopathology confirmed a diagnosis of primary cardiac myxofibrosarcoma.
Day 65	Contrast-enhanced CT revealed regrowth at the surgical site, consistent with local tumour recurrence.
Day 66	Head CT showed worsening of cerebral metastases with midline shift secondary to cerebral oedema.
Day 80	The patient was transferred to a hospice for palliative care.
Day 101	The patient passed away.

## Case presentation

**Figure 1:**
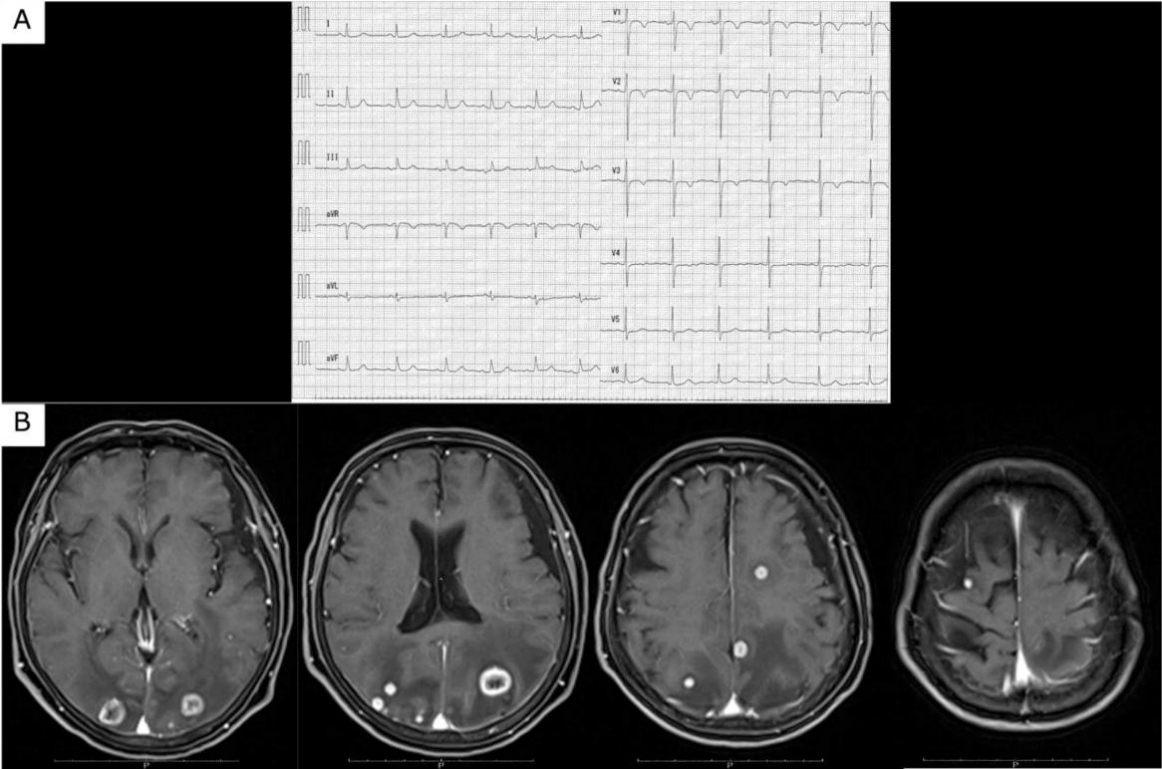
(*A*) Admission electrocardiography showing sinus rhythm and T-wave inversions in the lateral and anterior leads (augmented voltage left, V2–V4), consistent with non-specific repolarization abnormalities. (*B*) Contrast-enhanced brain magnetic resonance imaging reveals approximately 10 contrast-enhancing nodular and mass-like lesions located in the bilateral parietal and occipital lobes.

**Figure 2:**
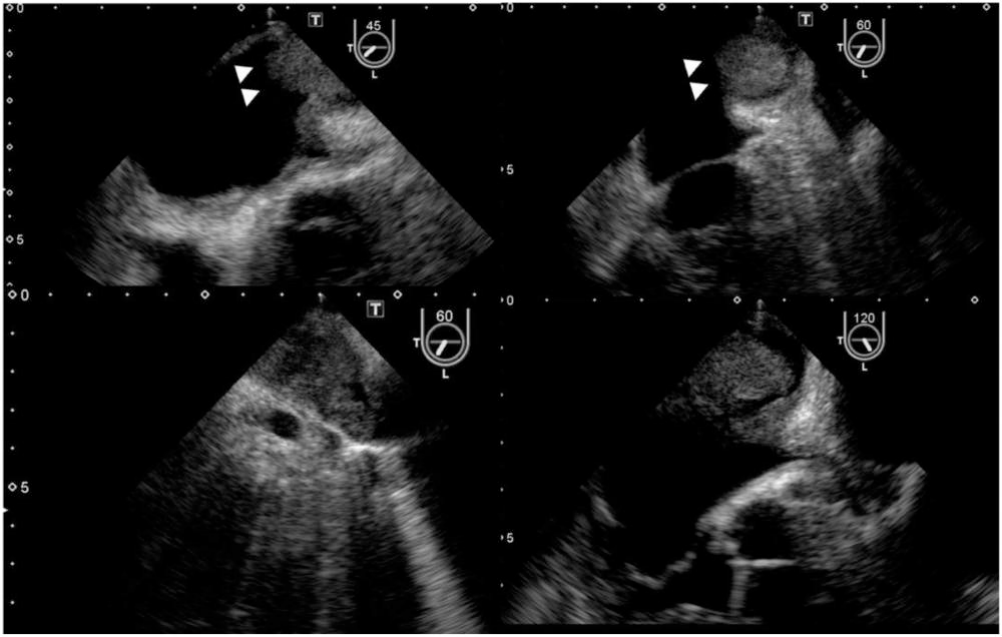
Transoesophageal echocardiography demonstrates a heterogeneous 40 × 25 mm mass obstructing the left pulmonary vein, with partial protrusion into the left atrial cavity (white arrows).

**Figure 3:**
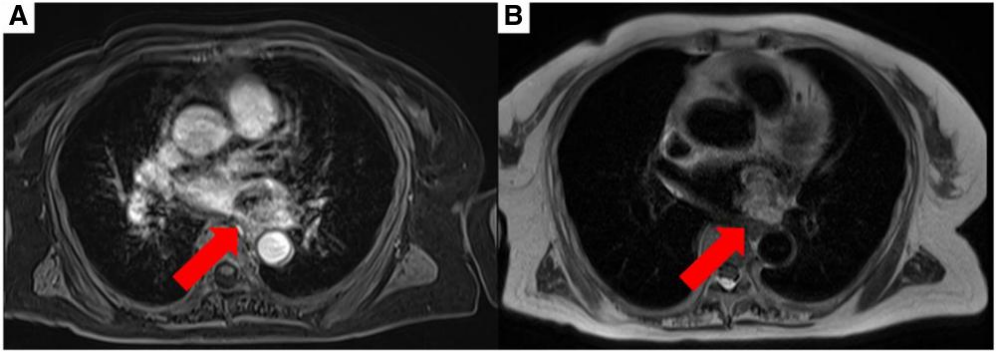
Contrast-enhanced cardiac magnetic resonance imaging findings. (*A*) T1-weighted imaging shows predominantly low signal intensity with areas of higher intensity. (*B*) T2-weighted imaging shows high signal intensity interspersed with low-intensity regions, suggesting internal heterogeneity.

**Figure 4:**
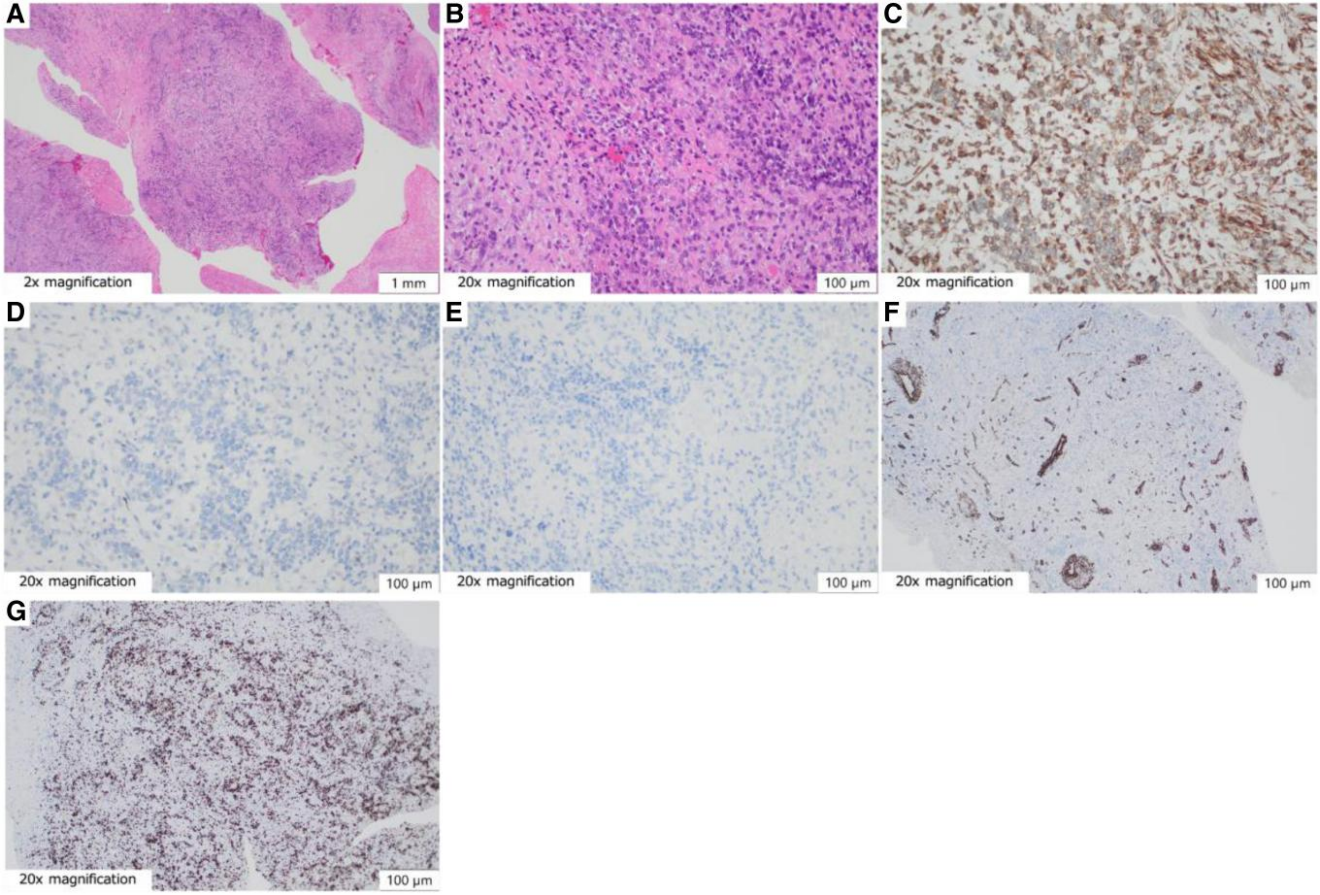
Histopathological and immunohistochemical features of the cardiac tumour. (*A*) Multinodular tumour architecture with fibrous septa and cleft-like spaces between nodules (haematoxylin and eosin staining). (*B*) Spindle- to polygonal-shaped cells exhibiting pronounced nuclear pleomorphism, nucleolar prominence, and abundant mitotic figures indicating active cell division. Myxoid stroma was interspersed between tumour cells, and regions with sparse nuclei suggested tumour necrosis (haematoxylin and eosin staining). (*C*) Vimentin immunostaining shows widespread cytoplasmic positivity in tumour cells, with nuclear pleomorphism and uneven cellular density, in line with the haematoxylin and eosin staining results. (*D*) Negative staining for S100, aiding in differential diagnosis exclusion, including malignant peripheral nerve sheath tumour or melanoma. (*E*) Negative staining for S100 and desmin further supports excluding malignant peripheral nerve sheaths and myogenic tumours. (*F*) Smooth muscle actin (SMA) staining is negative in tumour cells, with SMA positivity observed in the vascular smooth muscle and perivascular stromal regions, indicating non-neoplastic, structurally localized staining. (*G*) Immunohistochemical staining for Ki67 shows 70% positivity, suggesting a high tumour proliferative index.

**Figure 5:**
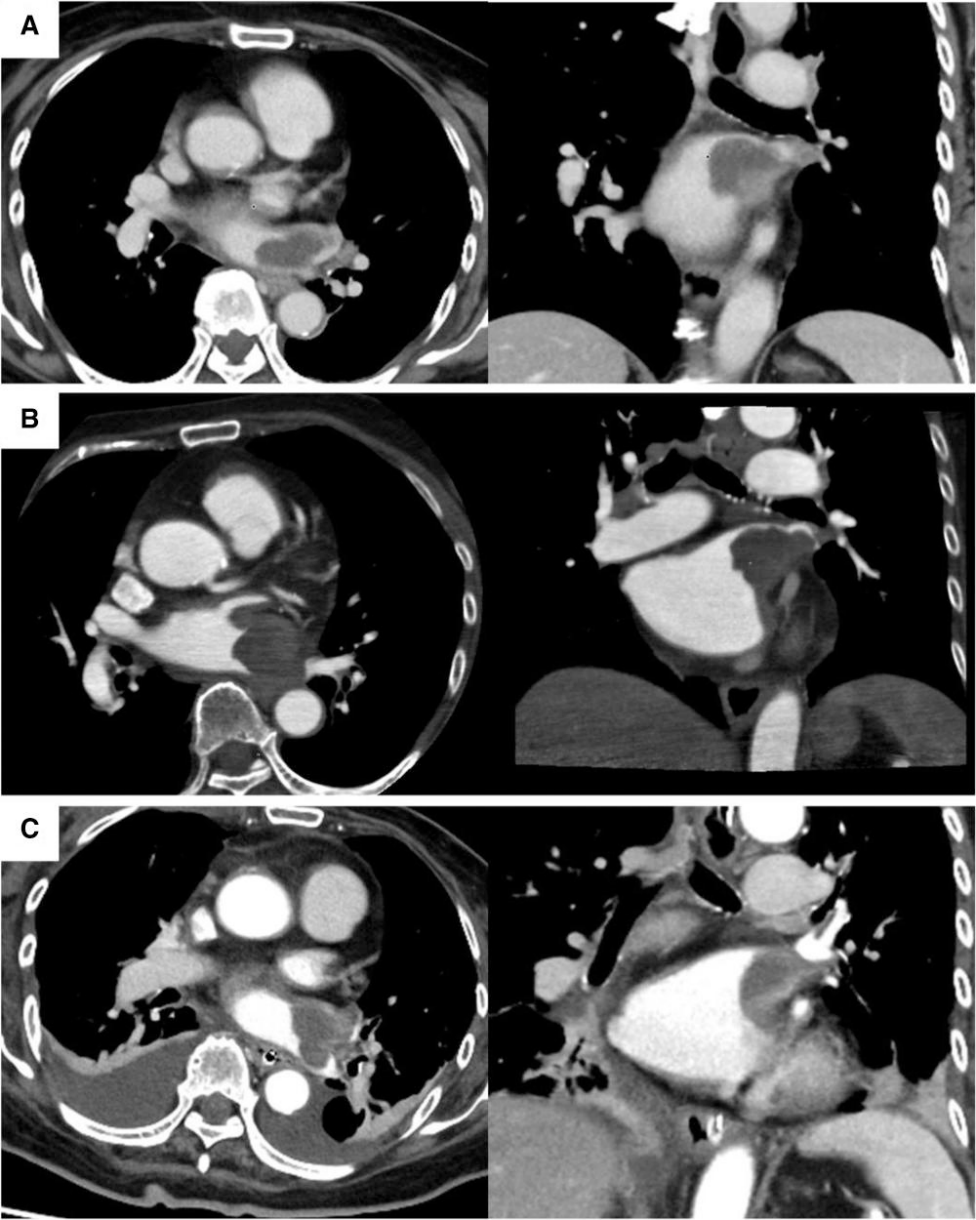
Contrast-enhanced computed tomography. (*A*) On Day 4, a contrast defect is visible in the left upper and lower pulmonary veins, with no primary tumours or lymphadenopathy detected elsewhere. (*B*) On Day 35, a broad-based exophytic mass obstructing the left pulmonary vein is visible, which appears larger than that in the previous imaging. (*C*) On Day 65, contrast-enhanced computed tomography shows tumour regrowth in the resected area, indicating local recurrence.

A 79-year-old woman presented with left-sided weakness and blurred vision. Brain imaging revealed cerebral oedema and multiple mass lesions in the bilateral parietal and occipital lobes, which prompted referral to our hospital for further evaluation and management. Her Glasgow Coma Scale (GCS) score was 14. Manual muscle testing showed bilateral weakness, worse on the right (Grade 4). Vital signs and chest auscultation were unremarkable. Blood tests showed elevated tumour marker levels, with a CA19-9 level of 40.5 U/mL. Chest radiograph showed cardiomegaly and the first right cardiac arc enlargement. Electrocardiography revealed sinus rhythm with T-wave inversions in leads augmented voltage left and V2–V4 (*Figure 1A*). A contrast-enhanced brain magnetic resonance imaging (MRI) revealed nodular and mass-like lesions with contrast enhancement at approximately 10 locations across the parietal and occipital lobes (*Figure 1B*). Suspecting brain metastasis, the trunk’s contrast-enhanced computed tomography (CT) on Day 4 revealed a contrast defect in the left pulmonary veins without primary tumours or lymphadenopathy. Echocardiography was conducted for suspected primary pulmonary vein tumour. Transthoracic echocardiography (TTE) on Day 18 showed no significant abnormalities, whereas transoesophageal echocardiography (TEE) on Day 21 showed a heterogeneous 40 × 25 mm mass, partially extended into the left atrium (*Figure 2*, Supplementary material online, *Video S1*). Contrast-enhanced cardiac MRI on Day 24 showed low signal intensity with some higher-intensity areas on T1-weighted images and predominantly high signal intensity with lower-intensity areas on T2-weighted images, suggesting tumour necrosis and haemorrhage indicating malignancy (*Figure 3*). On Day 35, contrast-enhanced cardiac CT revealed a broad-based exophytic mass obstructing left pulmonary vein, larger than before (see Supplementary material online, *Video S2*). The lesion was suspected to be malignant and to have originated in the pulmonary vein. Surgical extirpation was conducted on Day 57. Upon opening the right atrium and inspecting the left atrium, a tumour measuring 40 × 25 mm was found obstructing the left pulmonary vein. The tumour was primarily located in the left pulmonary vein and extended into the left atrium, consistent with a primitive cardiac tumour. The mass was fragmented because of the difficulty in removing *en bloc*. Histopathology revealed multinodular architecture with alternating hypocellular and hypercellular areas. Curved thin-walled vessels were scattered within myxoid stroma. The tumour comprised heterogeneous round and spindle-shaped cells with marked nuclear pleomorphism. Most tumour cells had mitosis and focal necrosis. Immunohistochemistry was positive for vimentin; focally positive for smooth muscle actin, CD31, D2–40, and factor VIII; and negative for S100, desmin, cytokeratin, and CD34. The Ki-67 index was approximately 70%, indicating high proliferation (*Figure 4*). The patient was diagnosed with primary cardiac myxofibrosarcoma. Contrast-enhanced CT on postoperative Day 8 revealed tumour regrowth in the resected area, which was considered to represent regrowth of residual tumour rather than local recurrence (*Figure 5*). The following day, head CT showed the progression of brain metastases and a midline shift due to oedema. The patient’s condition deteriorated, with the GCS score of 3. Consequently, postoperative chemotherapy and radiotherapy were considered unsuitable. A best supportive care approach was initiated, and the patient was transferred to a hospice on postoperative Day 23 (hospital Day 80), where she passed away 39 days post-transfer.

## Discussion

In 2015, the World Health Organization revised its classification of cardiac neoplasms to encompass benign and malignant tumours and tumour-like conditions.^5^ Cardiac tumours are categorized as primary or secondary. Primary and secondary tumours occur in approximately 0.05% and 1% of autopsy cases, respectively.^6,7^ Malignant neoplasms constitute approximately one-quarter of all primary cardiac tumours, 75%–95% of which are sarcomas, histopathologically.^1^ Clinical presentation varies, with a classical triad of obstructive, embolic, and systemic symptoms. The condition remains asymptomatic until advanced stages.^7^ In this case, neurological symptoms preceded the triad, possibly due to brain metastasis. Common imaging modalities include TTE, TEE, cardiac MRI/CT, and FDG-PET.^7–9^ TEE offers superior resolution for tumour extent and adjacent structures. Cardiac CT/MRI defines the tumour size, composition, vascularity, and spread. MRI differentiates benign from malignant lesions. Malignant characteristics include a tumour size of >5 cm, infiltration into multiple cardiac chambers, haemorrhagic pericardial effusion, exophytic growth with a wide base, and extension into the mediastinum.^10^ In this case, CT showed an exophytic mass with a broad attachment base, suggesting malignancy. Malignant cardiac tumours typically appear hypointense on T1-weighted and hyperintense on T2-weighted MRI, with heterogeneous signals due to necrosis and neovascularization. They also often show first-pass perfusion and late gadolinium enhancement, which helps distinguish them from benign tumours.^11^ Surgical resection is the primary treatment, with complete removal offering the best chance of prolonged survival. In advanced cases, palliative therapy may primarily alleviate symptoms.^6,7^ Brain metastases were present; however, resection was conducted to obtain a histological diagnosis before chemotherapy and radiotherapy. Histopathology confirmed the diagnosis of myxofibrosarcoma, a tumour with an aggressive biological behaviour. Local recurrence and distant metastasis were reported in up to 42.9% and 19.0% of cases, respectively, contributing to poor prognosis.^4^ In a review of 31 cases of primary cardiac myxofibrosarcoma reported in 24 publications, the mean tumour size was 43.1 mm, with the left atrium being the most frequently involved site (58.1%).^8^ Each tumour type favoured specific locations, consistent with these distribution patterns.^6^ Excluding cases where surgery was not conducted owing to locally advanced or metastatic disease and those lost to postoperative follow-up, the median survival time was 14 months. A tumour size of ≥40 mm and high histological grade were associated with poorer prognostic factors. The FNCLCC histological grade, based on differentiation, mitotic activity, and necrosis extent, also correlates with survival.^12^ In this case, the tumour measured ≥40 mm and was histologically categorized as high grade, which may explain the poor prognosis. Furthermore, an exophytic mass with a broad base makes complete resection difficult. Myxofibrosarcoma has a high local recurrence and distant metastasis rate, contributing to poor prognosis. Moreover, delayed surgical resection allows further progression and contributes to unfavourable outcomes. Given the rarity of these tumours, guidelines for primary cardiac tumours are lacking, and evidence on optimal diagnosis and treatment is limited. Reports have shown that chemotherapy and radiotherapy are more effective when combined with surgical resection.^8,13,14^ We verified the rapid progression of cardiac malignant tumours using multiple CT scans conducted over a short period, suggesting high-grade malignancy and intractable nature. Owing to the rapid progression of the disease, prompt evaluation using multiple imaging modalities is essential to distinguish between benign and malignant lesions, for early surgical intervention. Early surgery combined with chemotherapy and radiotherapy may be the only treatment available to improve clinical outcomes. Few reports have described aggressive treatment for this tumour with brain metastases, limiting the generalizability of the treatment and prognosis. Further case studies are required to guide the development of optimal management strategies.

## Lead author biography


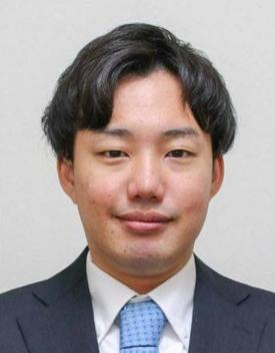


Koichi Nakamura is an interventional cardiologist and assistant professor in the Cardiovascular Medicine at Nippon Medical School Chiba Hokusoh Hospital. My research focuses on coronary artery disease.

## Supplementary Material

ytaf447_Supplementary_Data

## Data Availability

The data underlying this article are available in the article and in its online Supplementary material.

## Supplementary material

**Consent:** The authors confirm that written informed consent for the submission and publication of this case report, including associated images and clinical information, was obtained from the patient in accordance with the Committee on Publication Ethics guidelines.

**Funding:** None declared.
